# Formononetin: a promising therapeutic agent targeting the gut-lung axis in acute lung injury

**DOI:** 10.3389/fphar.2026.1761248

**Published:** 2026-06-04

**Authors:** Wuliji Batu, Daijian Liu, Chunxiu Ren, Wenhui Bi, Junmei Wang, Yu Wang

**Affiliations:** 1 Affiliated Hospital of Inner Mongolia Minzu University, Tongliao, Inner Mongolia, China; 2 Key Laboratory of Basic Pharmacology of Ministry of Education and Joint International Research Laboratory of Ethnomedicine of Ministry of Education, Zunyi Medical University, Zunyi, Guizhou, China; 3 School of Basic Medical Sciences, Inner Mongolia Minzu University, Tongliao, Inner Mongolia, China

**Keywords:** Acute lung injury, Parabacteroides, formononetin, gut-lung axis, gut microbiota

## Abstract

**Background:**

Acute lung injury (ALI) is a common acute inflammatory lung disease in critical clinical conditions, with a complex pathogenesis and limited available treatment options. Although the gut-lung axis has been identified as an emerging field of host-related microbiota in various chronic lung diseases, it remains unclear whether gut microbiota is associated with the development and progression of ALI.

**Objective:**

This study aims to investigate the therapeutic effects of formononetin on ALI mice and elucidate how formononetin ameliorates ALI by regulating the gut-lung axis.

**Methods:**

Pathological changes in lung and intestinal tissues of LPS-induced ALI mice were first examined using histopathological detection. Reverse transcription quantitative real-time PCR (RT-qPCR) and Western blot (WB) were employed to assess lung/intestinal inflammation and intestinal barrier integrity. 16S rRNA sequencing was used to evaluate the intestinal microbial microenvironment, and metabolomics was applied to detect changes in serum metabolites.

**Results:**

Formononetin effectively ameliorated lung injury in ALI mice, reduced the expression of lung/intestinal inflammatory factors, and protected the integrity of the lung-intestinal barrier in ALI mice. Gut microbiota analysis revealed that the anti-ALI effect of formononetin may be associated with Parabacteroides. Metabolite pathway enrichment involved bile acid metabolism and ABC transporters.

**Conclusion:**

Formononetin reverses lung injury and repairs the gut-lung barrier in ALI mice. These effects are accompanied by changes in Parabacteroides abundance, as well as alterations in ABC transporters and bile secretion. This study provides new insights and potential applications for the clinical intervention of ALI.

## Introduction

Acute lung injury (ALI) and its severe form, acute respiratory distress syndrome (ARDS), are life-threatening critical pulmonary conditions (Thille et al., 2013). While clinical outcomes for some patients have improved through optimized mechanical ventilation strategies, fluid management, as well as the use of prone positioning ventilation and extracorporeal membrane oxygenation (ECMO), ALI/ARDS remains one of the most common causes of mortality and disability in the intensive care unit (ICU), with a current mortality rate still as high as 35%–40% (Bellani et al., 2016). Currently, there is a lack of specific and proven effective pharmacological treatments. Management primarily relies on comprehensive symptomatic support, including mechanical ventilation, anti-infection therapy, anti-inflammatory measures, and nutritional support. The treatment process is prolonged, costly, and yields limited efficacy. Therefore, there is an urgent need to explore its mechanisms of action and identify targeted therapeutic drugs.

ALI arises from numerous pathogenic factors and involves intricate pathological processes, with its mechanisms not yet fully elucidated. However, accumulating evidence has confirmed that gut microbiota plays a crucial role in maintaining pulmonary health and immune system homeostasis. Dysbiosis of the gut microbiota contributes to the development of various lung diseases—such as pneumonia, tuberculosis, chronic obstructive pulmonary disease (COPD), bronchial asthma, idiopathic pulmonary fibrosis, and ARDS—by disrupting immune system function (Arrieta et al., 2015; Lai et al., 2022). Studies have found that a decrease in *Bifidobacterium* and an increase in *Clostridium* in the gut may promote immune responses dominated by respiratory allergies, mediating the development of asthma. The use of antibiotics to eliminate gut microbiota can lead to impaired pulmonary immune defense, increasing susceptibility to influenza virus and *Streptococcus pneumoniae*, reducing survival rates in mice, and exacerbating pulmonary inflammation and lung injury (Schuijt et al., 2015). Moreover, in patients with ARDS, bronchoalveolar lavage fluid has been found to be enriched with gut-associated microbiota (such as *Bacteroides*), and their abundance shows a significant correlation with the levels of systemic inflammatory cytokines. It is hypothesized that the occurrence of ARDS may be related to abnormal replication and translocation of intestinal microbiota into the lungs (Dickson et al., 2016). Fecal microbiota transplantation (FMT) has demonstrated significant therapeutic effects in a lipopolysaccharide (LPS)-induced acute lung injury (ALI) rat model by markedly reducing the lung wet/dry weight ratio and levels of pro-inflammatory markers (TNF-α, IL-1β, and IL-6), while simultaneously increasing arterial partial pressure of oxygen (PaO_2_) (Li B. et al., 2020). Furthermore, gut microbiota dysbiosis may play a regulatory role in host defense against LPS-induced ALI through the TLR4/NF-κB signaling pathway. Studies have found that intestinal flora imbalance may activate pulmonary oxidative stress and mediate lung injury by impairing the intestinal barrier. However, FMT restores the gut microecology, attenuates the activity of the TLR4/NF-κB signaling pathway in the lungs, and reduces oxidative stress in animal models of ALI (Tang et al., 2021). This underscores the significant role of the gut-lung axis in maintaining host homeostasis and influencing disease progression. Modulating the gut microbiota can markedly ameliorate the pathological state of ALI.

Formononetin, an isoflavone compound widely present in various plants, has been shown to possess multiple biological activities such as antioxidant, anti-inflammatory, and anti-tumor effects. In terms of its antioxidant properties, studies utilizing free radical scavenging assays and other methods have confirmed its ability to effectively decreased excessive free radicals in the body and reduce oxidative stress-induced cellular damage (Shi et al., 2015). Regarding its anti-inflammatory effects, some studies have found that formononetin can inhibit the activation of inflammation-related signaling pathways, such as the NF-κB pathway, thereby reducing the release of inflammatory factors and mitigating inflammatory responses (Huang et al., 2021). At the cellular level, formononetin exhibits multiple effects on cells relevant to acute lung injury (ALI). Studies have found that formononetin can inhibit the accumulation of reactive oxygen species (ROS) in lipopolysaccharide (LPS)-activated bronchial epithelial cells. By suppressing the activation of NADPH oxidase, it reduces the transcription and translation of inflammatory factors such as IL-6, CCL2, and CXCL8, thereby alleviating inflammatory responses (Li et al., 2024). Currently, the application of formononetin in the treatment of acute lung injury (ALI) remains in the preliminary research stage, and its specific mechanisms of action have not yet been fully elucidated. Therefore, it is necessary to further explore the anti-ALI mechanisms of formononetin from new perspectives.

By sequencing the host intestinal microbiota to analyze its composition and employing metabolomics to identify ALI-related biomarkers and mechanistic insights, the integration of metabolomics with microbiota analysis holds promise for elucidating the anti-inflammatory mechanisms of formononetin from the perspective of the gut-lung axis. This study utilized LPS to establish an ALI mouse model to investigate the anti-inflammatory effects of formononetin. The anti-inflammatory mechanisms of formononetin were further explored through combined gut microbiota and metabolomics experiments, thereby providing novel insights and a theoretical foundation for understanding its role in combating ALI.

## Materials and methods

### Animal experiments

This animal study was reviewed and approved by the Medical Ethics Committee of the Affiliated Hospital of Inner Mongolia Minzu University (Ethics Approval No.: NM-LL-2024-12-06-01). Fifty 8-week-old male SPF-grade BALB/C mice, weighing 18–22 g, were purchased from Liaoning Changsheng Biotechnology Co., Ltd. The mice were housed under the following conditions: temperature 20 °C–25 °C, relative humidity 45%–65%, 12 h automatic light-dark cycle, with free access to food and water. After 1 week of adaptive feeding, the experiment began. The mice were randomly divided into five groups (8 mice per group): blank control group, LPS model group (5 mg/kg), low-dose formononetin group (LPS + ForL, 25 mg/kg, twice daily), high-dose formononetin group (LPS + ForH, 50 mg/kg, twice daily), and dexamethasone group (LPS + DEX, 5 mg/kg, twice daily). During the experiment, mice in the For and DEX groups received continuous intraperitoneal injections of For or DEX, while the blank and model groups received corresponding solvents via intraperitoneal injection. After 7 days, all mice were exposed to LPS (5 mg/kg) via nasal inhalation for 1 h. Eight hours after LPS induction, all animals were anesthetized by intraperitoneal injection of sodium pentobarbital and euthanized. The right lung tissue and colon tissue of mice from each group were collected, rinsed with physiological saline, gently blotted dry with filter paper, weighed and recorded, air-dried in a cool and ventilated area, and weighed again. The lung wet weight/dry weight (W/D) ratio was calculated by comparing the two measurements. Pathological changes in lung and colon tissues were observed using hematoxylin-eosin (H&E) staining.

### Bronchoalveolar lavage fluid (BALF) cell analysis

A syringe was used to aspirate pre-warmed physiological saline, which was then injected into the mouse lungs for lavage. After 30 s, the lavage fluid was slowly withdrawn and reinjected. This step was repeated until the fluid appeared milky white and foamy. The BALF was collected and placed on ice, filtered through sterile gauze to remove mucus and impurities. The filtered lavage fluid was centrifuged at 4 °C and 500 × g for 10 min to collect the cell pellet. The pellet was resuspended in 0.5 mL of 0.9% sodium chloride solution to an appropriate concentration (5 × 10^6^/mL). The mixed BALF samples were centrifuged at 4 °C and 3000 rpm for 10 min, and the supernatant and cell pellet were separately aliquoted and stored for subsequent assays. The levels of IL-6 and TNF-α in the BALF were measured using ELISA kits (IL-6: BioLegend, USA; TNF-α: BioLegend, USA) according to the manufacturer’s instructions.

### qRT-PCR analysis of inflammatory factor expression in intestinal tissues

Total RNA was extracted from intestinal tissues using an RNA extraction kit (TIANGEN). The purity and concentration of RNA were analyzed using a NanoDrop 2000 spectrophotometer (D260/D280 > 1.8). Total RNA was reverse-transcribed into cDNA, and amplification was performed using the SYBR Green method under the following conditions: pre-denaturation at 95 °C for 10 min; 30 cycles of denaturation at 95 °C for 30 s, annealing at 60 °C for 30 s, and extension at 30 s. GAPDH was used as the internal reference, and the relative expression levels of IL-6, TNF-α, and IL-1β were calculated using the 2^−ΔΔCt^ method.

TNFα-F, 5′-CCC TCA CAC TCA GAT CAT CTT CT-3′; TNFα-R, 5′-GCT ACG ACG TGG GCT ACA G-3′; IL-6-F, 5′-ATG AAG TTC CTC TCT GCA AGA GAC T-3′; IL-6-R, 5′-CAC TAG GTT TGC CGA GTA GAT CTC-3′; IL-1β-F, 5′- GCA​ACT​GTT​CCT​GAA​CTC​AAC​T-3′; IL-1β- R, 5′-ATC​TTT​TGG​GGT​CCG​TCA​ACT-3′; GAPDH-F, 5′-CGA CTT CAA CAG CGA CAC TCA C-3′; GAPDH-R, 5′-CCC TGT TGC TGT AGC CAA ATT C-3′.

### Western blot analysis

Western blotting was widely used to detect protein expression levels. Mouse lung/intestinal tissues were cut into small pieces and placed in a mortar. Liquid nitrogen was added, and the tissues were continuously ground into freeze-dried powder. For every 50 mg of tissue, 1 mL of lysis buffer was added and incubated for 30 min. The lysate was centrifuged at 4 °C and 12,000 r/min for 10 min, and the supernatant was collected for BCA protein quantification. After determining the protein concentration, loading buffer was added, and the samples were heated in a 100 °C water bath for 5 min. Gels were prepared, samples were loaded, and electrophoresis was performed followed by membrane transfer. The membranes were blocked with 5% skim milk at room temperature for 2 h. Primary antibodies against IL-6 (1:1000), TNF-α (1:1000), and ACSL4 (1:100) were incubated overnight at 4 °C and then recovered. Secondary antibodies (1:5000) were incubated at room temperature for 1 h, followed by development and imaging. GAPDH was used as the internal reference.

### Gut microbiota analysis

After dissection, cecal contents from mice were collected into sterile EP tubes, rapidly frozen in liquid nitrogen, and transferred to a −80 °C freezer for storage. Total DNA was extracted using a DNA extraction kit. The hypervariable V3-V4 region of the bacterial 16S rRNA gene was amplified using primer pairs 338F (5′-ACT​CCT​ACG​GGA​GGC​AGC​AG-3′) and 806R (5′-GGACTACHVGGGTWTCTAAT-3′). PCR amplification was performed on an ABI GeneAmp® 9700 thermal cycler (ABI, California, USA). PCR products were purified by gel extraction, quantified, and sequenced on the Illumina MiSeq platform (Shanghai Majorbio Bio-Pharm Technology Co., Ltd.). Raw data were quality-controlled using Trimmomatic software and assembled with FLASH software. Clustering was performed using UPARSE software, and taxonomic annotation was conducted with the RDP classifier.

### Metabolomics analysis

After preparation, mouse serum samples underwent quality control (QC) checks. LC-MS analysis was performed using a Thermo Fisher UHPLC-Orbitrap Exploris 480 system (Shanghai Majorbio Bio-Pharm Technology Co., Ltd.). Chromatographic conditions: 3 μL samples were separated on an HSS T3 column (100 mm × 2.1 mm i.d., 1.8 μm) and detected by mass spectrometry. Mobile phase A consisted of 0.1% formic acid in water/acetonitrile (95/5, v/v), and mobile phase B consisted of 0.1% formic acid in acetonitrile/isopropanol/water (47.5/47.5/5, v/v/v). The flow rate was 0.40 mL/min, and the column temperature was 40 °C. Mass spectrometry conditions: data acquisition was performed in positive and negative ion scanning modes with a mass range of m/z 70–1050. Ion spray voltages were set to −3000 V for negative ions and 3500 V for positive ions, with sheath gas at 50 arb, auxiliary gas at 13 arb, ion source temperature at 450 °C, and collision energy cycled at 20–40–60 V. Raw data were processed using Progenesis QI software (Waters Corporation, Milford, USA) for baseline filtering, peak identification, integration, retention time correction, and peak alignment, resulting in a data matrix of retention time, m/z, and peak intensity. MS and MS/MS spectra were matched against public metabolite databases (HMDB: http://www.hmdb.ca/; Metlin: https://metlin.scripps.edu/) and the Majorbio in-house database for metabolite identification. Principal component analysis (PCA) and orthogonal partial least squares-discriminant analysis (OPLS-DA) were performed using the ropls package (Version 1.6.2) in R. Differential metabolites were annotated for metabolic pathways using the KEGG database (https://www.kegg.jp/kegg/pathway.html).

### Statistical analysis

Data were analyzed using GraphPad Prism 9. Measurement data are expressed as mean ± standard deviation (x̄ ± s). Differences between groups were assessed by one-way analysis of variance (ANOVA). A p-value <0.05 was considered statistically significant.

## Results

### Formononetin attenuates pulmonary inflammation in ALI mice

**FIGURE 1:**
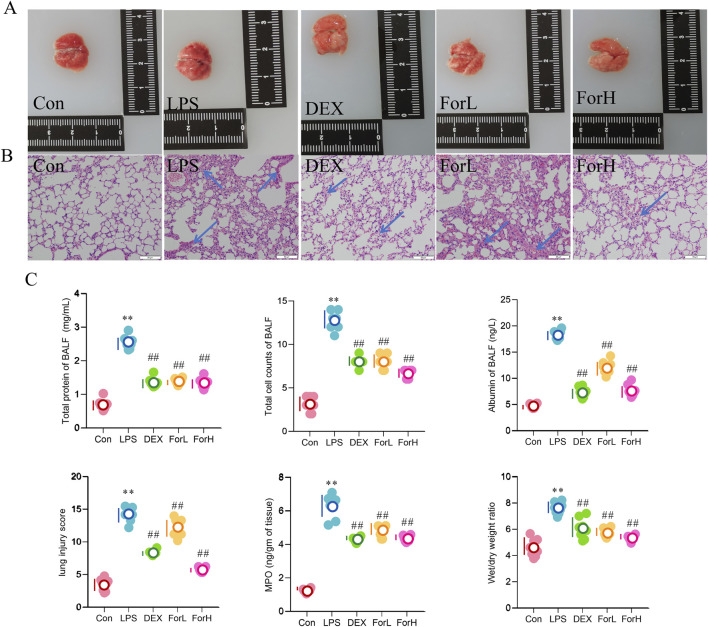
Formononetin alleviates lung tissue injury in ALI mice. **(A)** Macroscopic view of lungs; **(B)** HE staining results; **(C)** Total protein concentration, Total cell count and albumin in bronchoalveolar lavage fluid (BALF), Lung injury score, MPO and WD. **p < 0.01 vs. Con, ##p < 0.01 vs. LPS. n = 8.

An acute lung injury (ALI) model was established by exposing mice to LPS (5 mg/kg) via nasal inhalation for 1 h. The lung tissue of the control (Con) group exhibited normal morphology, smooth surface, and pink coloration, while the ALI group showed dark red hemorrhagic spots. In contrast, mice treated with formononetin (For) or dexamethasone (DEX) exhibited reduced hemorrhagic spots (Figure 1A). H&E staining revealed collapsed alveoli, significantly thickened interstitial septa, inflammatory cell infiltration, and obvious damage in ALI mice. Treatment with For or DEX markedly alleviated these LPS-induced histopathological changes, indicating that For and DEX could mitigate LPS-induced damage to lung tissue structure (Figures 1B,C). Quantitative analysis showed increased lung wet/dry weight ratio, total protein, total cell and alunmin in bronchoalveolar lavage fluid (BALF) in ALI mice, both of which were significantly reduced after For or DEX treatment (Figure 1C). Additionally, myeloperoxidase (MPO) activity assays demonstrated elevated MPO activity in the lung tissues of ALI mice, which was significantly reduced following For or DEX treatment (Figure 1C).

### Formononetin suppresses pro-inflammatory cytokine expression in ALI mice

**FIGURE 2:**
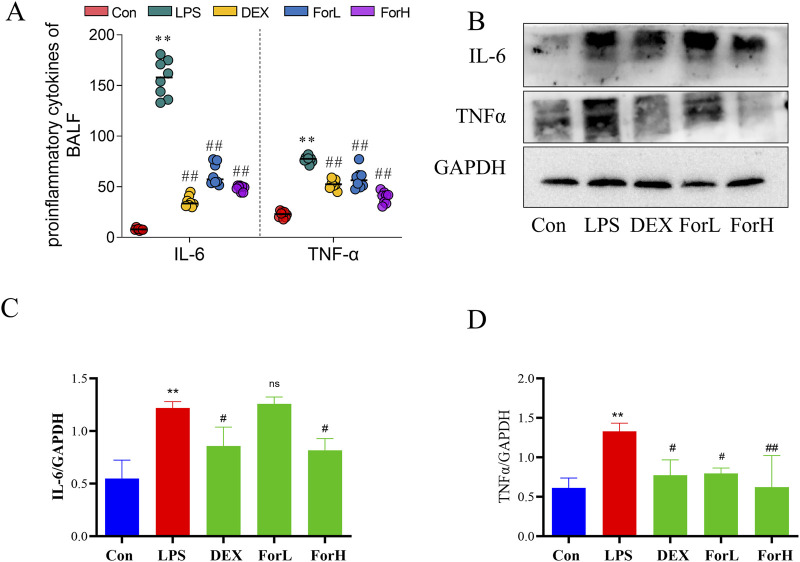
Formononetin inhibits the release of inflammatory factors in ALI mice. **(A)** Expression levels of proinflammatory cytokines IL-6 and TNF-ɑ detected by ELISA; **(B-D)** Expression of IL-6 and TNF-ɑ detected by Western blot. **p < 0.01 vs. Con, #p < 0.05, ##p < 0.01 vs. LPS. n = 3.

ELISA analysis of IL-6 and TNF-α levels in BALF revealed significantly elevated concentrations of these cytokines in ALI mice. Treatment with For or DEX markedly reduced IL-6 and TNF-α levels (Figure 2A). Concurrently, Western blot analysis confirmed that For or DEX treatment downregulated the expression of IL-6 and TNF-α in lung tissues of ALI mice (Figure 2B). Further protein quantitative analysis (Figures 2C,D) also confirmed this trend.

### Formononetin protects intestinal barrier integrity in ALI mice

**FIGURE 3:**
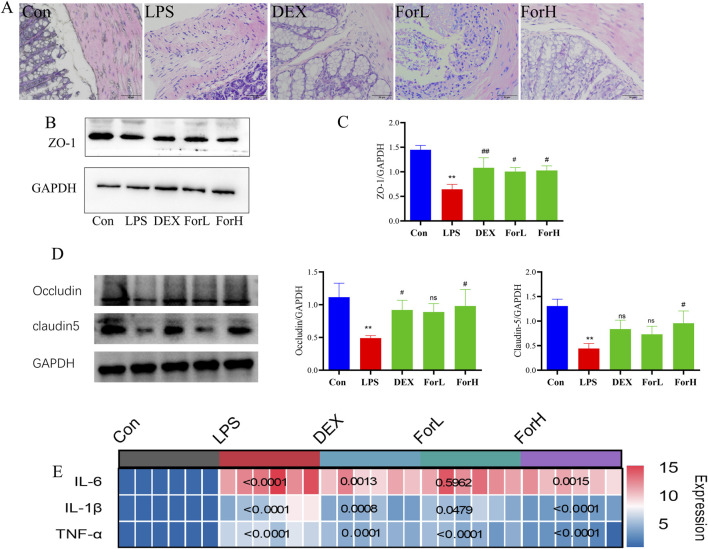
Formononetin decreases gut barrier leakage and downregulates the expression of inflammatory factors in ALI mice. **(A)** Colon HE staining; **(B-D)** Expression of ZO-1,Claudin-5 and Occludinin intestinal tissue detected by Western blot; **(E)** Expression of IL-1β, IL-6, and TNF-ɑ detected by RT-PCR. **p < 0.01 vs. Con, #p < 0.05, ##p < 0.01 vs. LPS. n = 3.

Studies have shown that the integrity of the intestinal epithelium plays a critical role in protecting the body against exogenous damage and antigen invasion (Qinghua et al., 2015). H&E staining demonstrated that For or DEX treatment ameliorated LPS-induced disruption of the intestinal mucosal barrier in mice (Figure 3A). Western blot analysis revealed a significant reduction in ZO-1,Claudin-5 and Occludinin protein expression in the colon of LPS-treated mice, which was restored after For or DEX intervention (Figures 3B–D). Furthermore, RT-qPCR results indicated elevated expression of IL-6, TNF-α, and IL-1β in the colon of ALI mice, which was suppressed by For or DEX treatment (Figure 3E).

### Formononetin modulates the “lung-gut” microbiota imbalance in LPS-Induced ALI mice

**FIGURE 4:**
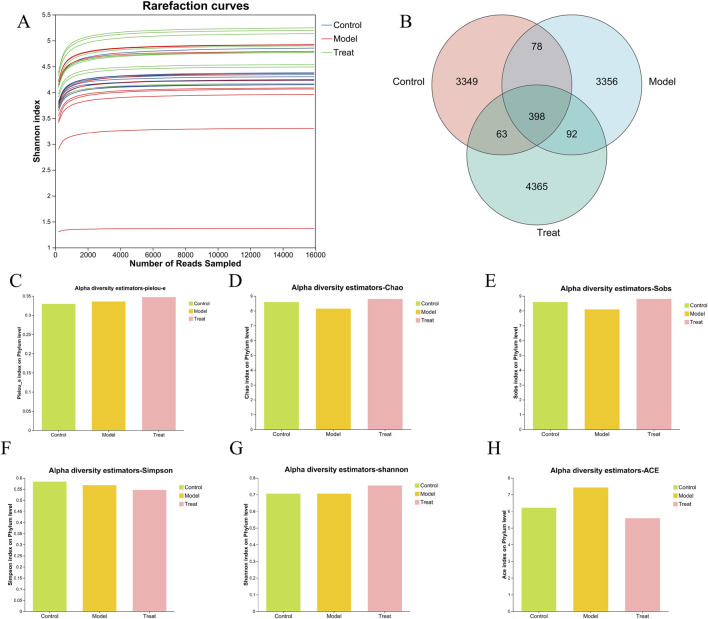
Formononetin alters LPS-induced gut-lung microbiota dysbiosis in ALI mice. **(A)** Rank-abundance curve; **(B)** ASV clustering Venn diagram; Microbial α-diversity evaluated by Pielou-e index **(C)**, Chao index **(D)**, Sobs index **(E)**, Simpson index **(F)**, Shannon index **(G)**, and ACE index **(H)**.

**FIGURE 5:**
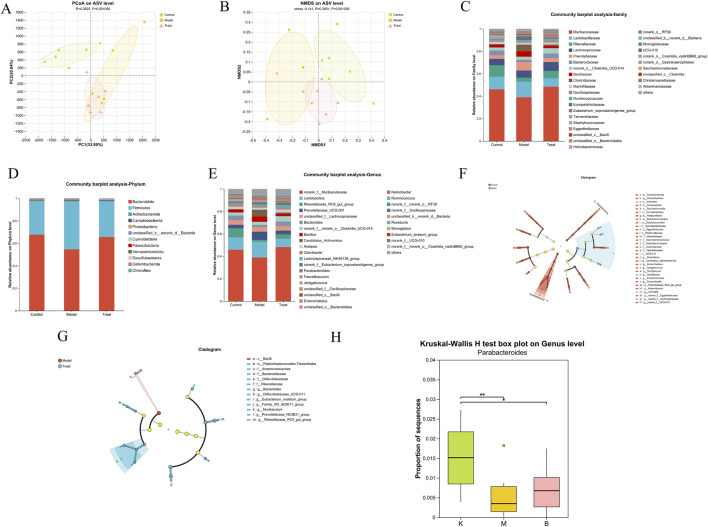
Formononetin modulates LPS-induced gut-lung microbiota dysbiosis in ALI mice. Microbial β-diversity assessed by PCoA **(A)** and NMDS **(B)**; Bacterial taxonomic analysis at phylum **(D)**, family **(C)**, and genus **(E)** levels; **(F)** LEfSe analysis between LPS group and Control group; **(G)** LEfSe analysis between LPS + ForH group and LPS group; **(H)** Statistical differences in Parabacteroides abundance at family level across three groups.

Clinical observations have revealed that patients with chronic lung diseases often exhibit intestinal microbiota imbalances. To determine whether For regulates the composition of the gut microbiota, we performed 16S rRNA sequencing on fecal samples from CON, LPS, and LPS + For groups of mice 8 h after LPS treatment. The rank-abundance curve showed that as sequencing depth increased, the number of ASVs gradually increased and eventually plateaued, indicating that the data volume was reasonable (Figure 4A). ASV clustering and species annotation of each sample were conducted, and the Venn diagram revealed that the disease altered the number of ASVs and modified the microbial community composition (Figure 4B). Figure 4D, E showed that the chao1 and Sobs indices indicated a reduction in the number of gut microbial species and a declining trend in species richness in LPS-induced ALI mice. The pielou-e, Shannon, Simpson, and ACE indices demonstrated that after For treatment, microbial richness decreased while evenness increased, and diversity indices improved, suggesting a trend toward microbiota optimization (Figures 4C,F–H). Principal coordinate analysis (PCoA) and non-metric multidimensional scaling (NMDS) revealed that the disease altered the structure of the gut microbiota in mice, while drug treatment modulated the composition and diversity of the gut microbiota in model mice, providing a protective effect against gut microbiota disruption (Figures 5A,B). Figure 5D presented an analysis at the phylum level, showing that compared to the control group, the abundance of Bacteroidetes in the intestinal tissue of LPS group mice significantly decreased, while the abundance of Firmicutes increased. For treatment markedly reversed these changes, bringing the microbial abundance in the intestinal tissue closer to that of the control group. We also conducted analyses at the family and genus levels. At the family level, results showed that compared to the control group, the abundance of Muribaculaceae and Rikenellaceae decreased in the intestinal tissue of LPS group mice, while the abundance of Lachnospiraceae and Prevotellaceae increased. After For treatment, the abundance of these microbial families was altered and brought closer to that of the control group (Figure 5C). At the genus level, results indicated that compared to the control group, the abundance of *Bacteroides* decreased in the intestinal tissue of LPS group mice, while the abundance of *Lactobacillus* and *Bacillus* increased. ForH treatment modified the abundance of these microbes and brought them closer to the control group levels (Figure 5E). LEfSe analysis of differential bacteria with an LDA score threshold of two revealed that at the family level, Lachnospiraceae was more abundant in the LPS group compared to the control group, consistent with the trend in species composition analysis. Compared to the LPS group, Rikenellaceae, Bacteroidaceae, and Anaeroplasmataceae were more abundant in the LPS + For group (Figures 5F,G). These results suggest that For significantly modulates the composition of the gut microbiota in LPS-induced ALI mice. Statistical analysis of differences in Parabacteroides at the family level across the three groups showed enrichment in the LPS + For group, which may be related to the drug’s therapeutic effects (Figure 5H).

### Formononetin alters the biological metabolism of LPS-Induced ALI mice

**FIGURE 6:**
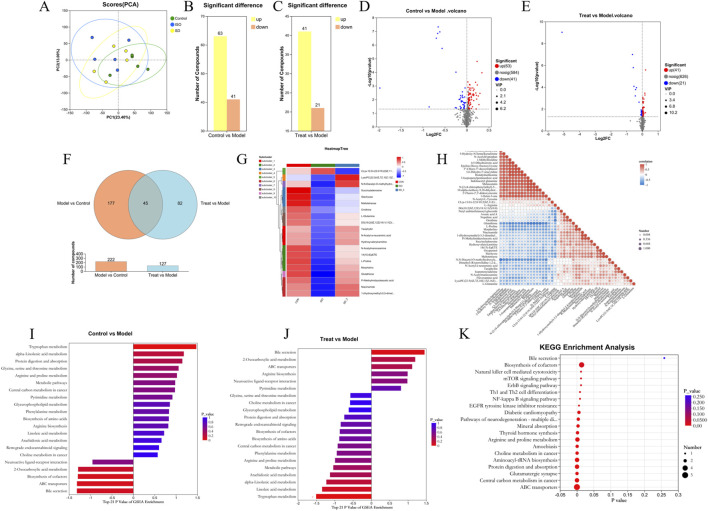
Formononetin modulates metabolic profiles in LPS-induced ALI mice. **(A)** PCA analysis; **(B)** Number of metabolites in LPS vs. Control group; **(C)** Number of metabolites in LPS + For vs. LPS group; **(D)** Volcano plot of LPS vs. Control group; **(E)** Volcano plot of LPS + For vs. LPS group; **(F)** Venn diagram of metabolites; **(G)** Clustering heatmap of metabolites; **(H)** Correlation heatmap of metabolites; **(I)** GSEA enrichment of LPS vs. Control group; **(J)** GSEA enrichment of LPS + For vs. LPS group; **(K)** KEGG enrichment analysis of metabolites.

Given the regulatory effect of For on the gut microbiota, we subsequently conducted metabolomic analysis. Figure 6A displays the distribution trends of different groups, with samples from the CON, LPS, and LPS + For groups exhibiting distinct metabolic profiles, indicating differences. Compared to the control group, LPS group mice showed significantly altered levels of metabolites, while For treatment notably restored the distribution of these metabolites (Figures 6B,C). Compared to the control group, the LPS group had 63 increased differential metabolites and 41 decreased differential metabolites (Figure 6D). However, compared to the LPS group, the LPS + For group had 41 increased differential metabolites and 21 decreased differential metabolites (Figure 6E). Additionally, 45 common differential metabolites were observed in both comparisons. Cluster heatmap analysis of the differential metabolites showed clear separation in each comparison (Figures 6F,G), while a triangular heatmap displayed the correlations among these 45 differential metabolites (Figure 6H). Subsequent GSEA enrichment results of the differential metabolites revealed that most differential metabolites between the control and LPS groups exacerbated disease expression through downregulated pathways such as ABC transporters, bile secretion, 2-oxocarboxylic acid metabolism, and neuroactive ligand-receptor interactions. However, after drug treatment, the regulation of these pathways was reversed (Figures 6I,J). Furthermore, KEGG enrichment analysis of the differential metabolites found that the metabolic pathways were associated with ABC transporters and bile secretion (Figure 6K).

## Discussion

Acute lung injury (ALI) is a severe disease with high incidence in intensive care units (ICUs) (Du et al., 2014). A study of 1,149 critically ill patients found that 368 (32%) developed acute respiratory distress syndrome (ARDS, the severe stage of ALI) within the first 4 days of ICU admission (Chen et al., 2015). Although progress has been made in understanding its molecular mechanisms and pharmacological treatments, the exact pathogenesis remains unclear.

Respiratory diseases are closely linked to gastrointestinal disorders. The gut microbiota plays a critical role in immune adaptation and initiation, influencing not only the gastrointestinal tract but also distal mucosal sites such as the lungs (Samuelson et al., 2015). Studies have shown that gut dysbiosis is associated with the development and progression of pulmonary diseases, including asthma and chronic obstructive pulmonary disease (Samuelson et al., 2015; Shima et al., 2016). Community-acquired pneumonia involves both intracellular and extracellular bacteria, and alterations in the gut microbiota may affect lung infections. For example, changes in nutrient availability, oxygen levels, pH, and interferon-γ can directly impact the life cycle and clearance of *Chlamydia*, though data on the gut microbiota’s influence on *Chlamydia* infection are still limited (Shima et al., 2016). Metabolomic studies of children with recurrent respiratory infections revealed differences in metabolic profiles compared to healthy children, with some metabolites reflecting variations in gut microbiota. Although treatment with pidotimod improved metabolic profiles, differences in gut microbiota-related metabolites persisted, suggesting a potential role of gut microbiota in recurrent respiratory infections (Bozzetto et al., 2017).

Thus, we hypothesized whether modulating the microbiota between distal organs and the lungs could influence disease progression. This study found that LPS induction caused significant gut microbiota disruption and systemic inflammation in mice, indicating that dysbiosis accelerates ALI progression by promoting systemic inflammation.

In this study, the 16S rRNA gene sequencing showed that bacteroidota was the microbial community with significant changes in abundance after formononetin treatment. Previous studies have confirmed that bacteroidota is the main source of intestinal bile salt hydrolase (BSH) and ABC transporter, and its abundance directly regulates bile acid metabolism and bile secretion pathway (Shang et al., 2025). Based on the “gut liver axis” regulation theory, intestinal flora disorder can lead to the downregulation of ABC transporter expression and abnormal bile secretion, and the restoration of beneficial flora abundance can help reverse the inhibition of this pathway, promote bile acid excretion and reduce inflammatory injury (Deng et al., 2026). In this study, formononetin treatment recovered bacteroidota and improved ABC transport and bile secretion, which was consistent with the above mechanism. We further explored the related strains after formononetin treatment, and found that parabacteroides in bacteroidea was the most relevant. It is worth noting that the study published in immunity confirmed that parabacteroides merdae in the intestine is the key strain to produce endogenous formononetin, and its abundance change can directly regulate the inflammatory state of the body (Chen et al., 2023). This finding is consistent with the results of this study that formononetin treatment increased the abundance of parabacteroides and alleviated inflammation, suggesting that parabacteroides may be the key mediating bacteria for formononetin to play an anti-inflammatory role.

**FIGURE 7:**
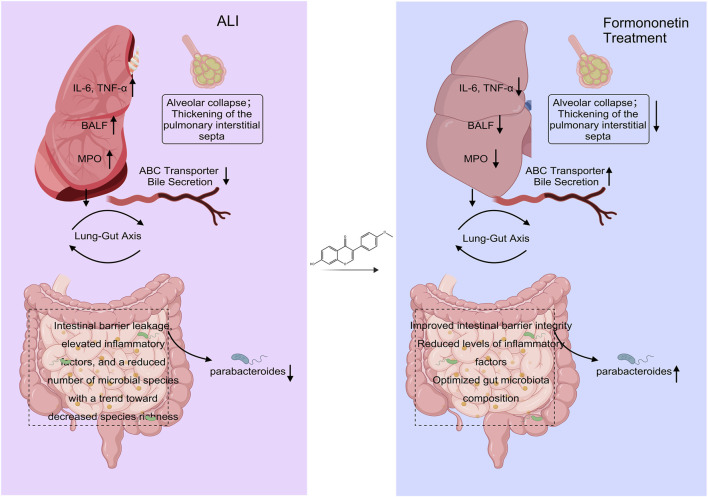
Formononetin attenuates lung injury and restores gut-lung barrier integrity in ALI mice by modulating Parabacteroides abundance, thereby influencing ABC transporters and bile secretion.

In our study, formononetin reversed these changes and restored the gut-lung barrier, potentially by regulating the abundance of *Parabacteroides* and subsequently influencing ABC transporters and bile secretion (Figure 7). We are the first to demonstrate that formononetin alleviates LPS-induced lung and intestinal injury, providing experimental evidence that the “gut microenvironment—including the intestinal barrier and gut microbiota”—serves as a novel target for formononetin in ameliorating inflammation and metabolic disorders in ALI mice.

Studies on ulcerative colitis (UC) and concurrent pulmonary inflammation have reported alterations in the abundance of *Parabacteroides* and other genera in feces, suggesting that *Parabacteroides* influences the development and progression of ALI (Li et al., 2016). In a rat model of sepsis-induced ALI, sepsis-induced ALI was associated with the abundance of potentially pathogenic bacteria such as *Parabacteroides* in the gut, indicating that modulating *Parabacteroides* and related genera may have therapeutic potential for sepsis-associated ALI (Han et al., 2023). Research on severe acute pancreatitis-associated ALI (SAP-ALI) also showed regulation of *Parabacteroides* relative abundance (Zhang et al., 2015). This study found a significant decrease in *Parabacteroides* abundance in the feces of ALI mice, while formononetin treatment markedly increased its abundance. Concurrently, metabolomics revealed that ABC transporters and bile secretion are closely related to the anti-ALI effects of formononetin.

In mammalian lungs, ABC transporters such as ABCA1, ABCG1, and ABCA3 are involved in the transport of cholesterol and phospholipids from lung cells and are crucial for maintaining pulmonary lipid homeostasis. Studies have shown that impaired activity of these ABC transporters leads to lipid accumulation and elevated levels of inflammatory cytokines in lung tissue, causing signs of respiratory distress in mice. This suggests that ABC transporters play a protective role in the lungs, and their dysfunction may contribute to ALI development (Chai et al., 2017). Bile acids also significantly impact ALI. In a rat model of LPS-induced ALI, ursodeoxycholic acid (UDCA) and chenodeoxycholic acid (CDCA) alleviated endotoxin-induced lung injury by modulating the expression of aquaporins 1 and 5 (AQP1 and AQP5), reducing oxidative stress, regulating apoptotic pathways (BAX, caspase 3, BCL-2), and attenuating the pro-inflammatory activity of nuclear factor kappa B (NF-κB). This indicates that bile acids may protect against ALI through multiple mechanisms (Milivojac et al., 2025). Additionally, the organic solute transporter OSTα-OSTβ protects ileal epithelial cells from bile acid-induced damage, and its dysfunction may affect bile acid transport and metabolism, indirectly influencing ALI progression (Ferrebee et al., 2018).

In studies of neonatal cholestatic disease (NCD), differences in the relative abundance of *Parabacteroides* and other genera were observed between healthy infants and those with NCD, and these differences were associated with bile acid metabolism. This suggests that *Parabacteroides* may participate in bile acid metabolic processes, which are closely linked to ABC transporters (e.g., ABCG5 and ABCG8 involved in cholesterol transport in bile acids). Thus, *Parabacteroides* may indirectly affect ABC transporter function by influencing bile acid metabolism (Li M. et al., 2020). Research on gut microbiota and metabolomes of infants delivered via different methods found that cesarean section infants had lower abundance of *Parabacteroides* and their metabolomes were positively correlated with ABC transporter pathways, indicating that delivery mode may influence ABC transporter-related metabolic processes by affecting *Parabacteroides* and other genera (Li N. et al., 2020).

Due to experimental constraints, fecal microbiota transplantation (FMT) was not performed in this study. In future research, we will transplant the feces of formononetin-treated mice into Germ-free mice to directly validate the necessity and sufficiency of the gut microbiota in the anti-inflammatory effects of formononetin.

In conclusion, supplementation with *Parabacteroides* and related bacteria can halt the progression of lung injury in ALI mice. The anti-ALI effects of formononetin may be associated with *Parabacteroides*. This study reveals that formononetin treatment is associated with alterations in the abundance of Parabacteroides, as well as changes in bile acid metabolism and ABC transporters, suggesting its potential involvement in ALI treatment. These findings provide new insights and potential therapeutic strategies for clinical intervention in ALI.

## Data Availability

The original contributions presented in the study are publicly available. This data can be found here: https://figshare.com/s/6eaa5e70ffc7bd7e2200.
